# Factors influencing postpartum depression among Japanese parents: A prospective longitudinal study

**DOI:** 10.1002/npr2.12326

**Published:** 2023-03-13

**Authors:** Yuko Yamakawa, Michio Maruta, Yuya Higuchi, Akiko Tokunaga, Ryoichiro Iwanaga, Sumihisa Honda, Akira Imamura, Goro Tanaka

**Affiliations:** ^1^ Department of Occupational Therapy Sciences Nagasaki University Graduate School of Biomedical Sciences Nagasaki Japan; ^2^ Department of Health Sciences Nagasaki University Graduate School of Biomedical Sciences Nagasaki Japan; ^3^ Hizen Psychiatric Center Saga Japan; ^4^ Department of Nursing Sciences Nagasaki University Graduate School of Biomedical Sciences Nagasaki Japan

**Keywords:** father and mother, parent report, parenting stress, postpartum depression symptoms, stress coping skills

## Abstract

**Aim:**

Postpartum depression (PPD) may have negative effects on the parents and lead to impaired cognitive, socioemotional, and behavioral development in their children. The purpose of this study was to examine factors associated with PPD in parents during the first year after delivery.

**Methods:**

This study used a self‐administered questionnaire. Questionnaires were mailed at 5 days, 3 months, 6 months, and 1 year after delivery, respectively. The particpants were 107 pairs of mothers and fathers. PPD was assessed using the Edinburgh Postnatal Depression Scale (EPDS). Data on sense of coherence (SOC), Quality Marriage Index, Social Support Scale, Mother‐to‐Infant Bonding Scale, and sociodemographic variables were collected. Multiple regression analysis was performed to examine the strength of the association between several variables and the EPDS at each survey period for fathers and mothers, respectively.

**Results:**

The prevalence of PPD in the first‐year postpartum ranged from 12.1%–23.4% to 7.5%–8.4% for fathers and mothers, respectively. SOC had the strongest impact on EPDS scores for both fathers and mothers at all four survey periods.

**Conclusions:**

Our findings suggest that stress coping skills are an important factor affecting PPD throughout the first‐year postpartum for both fathers and mothers.

## Abstract 2

We examined factors associated with postpartum depression (PPD) in parents during the first year after delivery. We found that Sense of Coherence had the most substantial impact on PPD scores for both fathers and mothers during all four survey periods (a few days, 3 months, 6 months, and 1 year after delivery) conducted during the first year. Our findings suggest that stress‐coping skills are an essential factor affecting PPD throughout the first year postpartum for both fathers and mothers.

## Graphical abstract

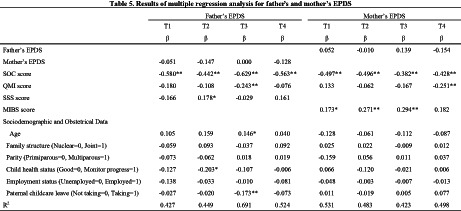

## 1 INTRODUCTION

Postpartum depression (PPD) refers to a depressive episode that occurs during pregnancy and the first year after delivery.^1^ PPD has been described primarily in mothers; however, in the past two decades, several studies have focused on PPD among fathers as well. A recent meta‐analysis reported the prevalence of maternal PPD to be 5.0%–26.3%, depending on the country.^2^ Another meta‐analysis reported an 8.8% prevalence of PPD in fathers during the first‐year postpartum.^3^ The prevalence of PPD during the first year postpartum in Japan ranged from 8.2%–13.2% to 11.5%–15.1% for fathers and mothers, respectively.^4^, ^5^ The presence of PPD affects marital disharmony and family functioning^6^ and may also lead to impaired cognitive, socio‐emotional, and behavioral development and mental health in their children.^7^, ^8^ Children of mothers with subclinical depressive symptoms may also experience their negative effects, as do children of mothers with clinical depression.^9^, ^10^ Therefore, PPD is a global public health concern^11^ and also a key issue in the “Healthy Parents and Children 21 (2nd Phase)” initiative being promoted by the Japanese Ministry of Health, Labor and Welfare (MHLW).^12^

Implementing a targeted and effective strategy requires understanding of factors that influence PPD. Several studies have identified various risk factors for maternal PPD.^2^, ^13^, ^14^, ^15^ The available evidence on risk factors associated with PPD in fathers is also growing.^16^, ^17^ While the previous study on PPD has identified various factors, sorting out modifiable factors, including psychosocial factors, may help improve postpartum outcomes for parents. For instance, sense of coherence (SOC) has been reported as a risk factor for PPD, but most reports have focused on mothers,^18^, ^19^, ^20^, ^21^ and few have included fathers.^22^, ^23^ Mother‐infant bonding may affect maternal mental health after childbirth.^24^, ^25^ There is also growing evidence available on risk factors associated with PPD in fathers (e.g., unemployment, low social support, etc.).^16^, ^17^ However, given the evidence of an association between paternal and maternal depressive symptoms during pregnancy and after delivery,^26^ it is not sufficient to identify either paternal or maternal factors.^16^ Longitudinal studies investigating factors associated with PPD involving both parents are needed to construct effective preventive strategies. Previous longitudinal studies that included both parents have shown that mother‐in‐law and daughter‐in‐law relationship satisfaction, mutual depression, and marital relationship satisfaction are related to PPD.^27^, ^28^ A previous study of married couples in Japan reported that history of mental health disorders, household income, infant disease under medical treatment, partner depression, and marital satisfaction were associated with PPD in fathers.^17^, ^29^ However, their study conducted in Japan investigated only one (after 4 months) or two (after 1 month and 6 months) time periods between 1 and 6 months postpartum. Considering the possibility that PPD may occur during the first year after delivery, a longitudinal investigation of at least 1 year postpartum is warranted.

Longitudinal studies including fathers and mothers simultaneously on factors affecting PPD are limited, especially in the Japanese context. Furthermore, previous studies that have longitudinally examined factors affecting PPD indicate that these factors may vary by postpartum time. Therefore, this study aimed to conduct a longitudinal survey from a few days to 1 year after delivery to investigate factors affecting PPD in fathers and mothers at each survey period (a few days, 3 months, 6 months, and 1 year after delivery). We also investigated whether psychosocial factors in the few days postpartum predicted PPD in subsequent periods.

## 2 METHODS

### 2.1 Participants and procedure

This prospective longitudinal study investigated factors related to PPD in parents from a few days to 1 year after delivery. Between September 2011 and February 2013, 1276 couples who had given birth at a local obstetric clinic in urban Kyushu, Japan, were enrolled. The clinic was staffed by five full‐time obstetricians, several part‐time obstetricians, and 25 nursing personnel (midwives, nurses, assistant nurses, and nursing assistants), who played a central role in the development of the local child delivery environment. Through collaboration with a perinatal medical center that provides advanced and specialized medical care, the clinic offers a safe perinatal care system for women with high‐risk pregnancies. The clinic also offers 24‐hour telephone consultation to expectant and postpartum mothers, health checkups, and telephone follow‐ups at 2 weeks and 1 month postpartum, classes on breast massage and nipple care for postpartum mothers, and access to a postpartum care center (established in 2014). We distributed self‐report questionnaire to couples who gave consent at a few days (T1), 3 months (T2), 6 months (T3), and 1 year (T4) after delivery, respectively. The questionnaire included an enclosed reply envelope; thus, the responses were collected via mail. The questionnaire explained to participants the purpose of the study and that the information collected would be kept confidential and used only for the purposes of this study. By answering the questions in the questionnaire, the respondents reaffirmed their consent to the study. Our study protocol was approved by the Saga University (Faculty of Medicine) Ethics Committee (Ref. Nos. 23–60).

### 2.2 Measures

#### 2.2.1 Postpartum depression symptoms

PPD was measured using the Japanese version of the Edinburgh Postnatal Depression Scale (EPDS).^30^, ^31^ The EPDS is a self‐rated questionnaire designed to screen for symptoms of emotional distress during pregnancy and postpartum period,^30^ with validity and reliability verified for the Japanese version.^31^ The scale consists of 10 items, each graded on a scale from 0 to 3, with a total score range of 0–30. Mothers who score 9 or higher are considered to have depressive symptoms, and this cutoff score showed a sensitivity of 75% and a specificity of 93%.^31^ Whereas fathers with scores of 8 or higher are considered to have depressive symptoms.^17^ Since it is generally believed that men may be less expressive about their feelings, a lower cutoff score is applied than the cutoff score used for mothers.^32^ The Cronbach's α of the EPDS for each period in the current sample was 0.71–0.82 and 0.75–0.83 for fathers and mothers, respectively.

#### 2.2.2 Sense of coherence

We used the Japanese version of the SOC questionnaire, which consists of the 13 items.^33^ Participants rated their level of agreement with each question on a 7‐point Likert scale, with a total score ranging from 13 to 91. Higher total scores reflect stronger SOC. A previous study validated the Japanese version of the scale.^34^ Permission to use SOC is granted through the Society for Theory and Research on Salutogenesis (STARS).^35^ The Cronbach's α of the SOC for each period in the current sample was 0.85–0.90 and 0.88–0.91 for fathers and mothers, respectively.

#### 2.2.3 Marital satisfaction

Marital satisfaction was assessed using the Quality Marriage Index (QMI).^36^, ^37^ The questionnaire consists of the six items, and participants rate their level of agreement with each item on a 4‐point Likert scale. A high QMI score indicates that the marriage partnership is viewed favorably. The Cronbach's α of the QMI for each period in the current sample was 0.87–0.94 and 0.90–0.96 for fathers and mothers, respectively.

#### 2.2.4 Social support scale (SSS)

We assessed social support for fathers at the workplace using the subscales of the Brief Job Stress Questionnaire (BJSQ).^38^ The questionnaire was developed by the MHLW and is widely used for stress check program in Japanese companies.^39^ The BJSQ consists of 57 items, 9 of which measure social support, including support from superiors, co‐workers, family, and friends. Participants rate their level of agreement with each item on a 4‐point Likert scale. Higher total scores reflect lower levels of support. The reliability and validity of the BJSQ have been verified.^40^ The Cronbach's α of the social support scale for each period in the current sample was 0.85–0.87.

#### 2.2.5 Maternal–infant bonding

The Mother‐to‐Infant Bonding Scale (MIBS) was used to assess bonding between mother and infant.^41^ This scale was originally developed as a 9‐item questionnaire^41^ and was modified to 10 items in the Japanese version.^42^ Participants are asked to rate the each items on a 4‐point Likert scale from 0 to 3. The higher scores reflect worse mother‐to‐infant bonding. The Cronbach's α was 0.57–0.70 for the current study.

#### 2.2.6 Sociodemographic and obstetrical data

Data on sociodemographic variables, including paternal age (years), maternal age (years), family structure (“nuclear” or “joint”), employment (“yes” or “no”), and paternal childcare leave (“yes,” “planning to take,” or “no”) were collected. We also obtained information on parity (“primiparous” or “multiparous”) and the child health status (“good” or “monitor progress”).

### 2.3 Statistical analysis

Descriptive statistics were used to calculate participant characteristics of sociodemographic and obstetric data and the proportion of PPD at each survey period for fathers and mothers, respectively. Repeated‐measures analysis of variance with the Bonferroni correction was used to compare each assessment scale by survey period. Pearson's correlation analysis was used to investigate the association of various factors with the EPDS. Multiple regression analysis with the EPDS as the dependent variable was also performed to examine the strength of the association between several variables and the EPDS at each survey period for fathers and mothers, respectively. The explanatory variables included sociodemographic and obstetrical data, SOC, QMI, SSS on the BJSQ, and MIBS. We also performed multiple regression analysis to determine whether explanatory variables in the initial few days postpartum predict subsequent EPDS (T2, T3, and T4). All statistical analyses were performed using IBM SPSS Statistic version 25.0 (IBM Corp., Armonk, NY). Statistical significance was set at *p* < 0.05 all tests.

## 3 RESULTS

### 3.1 Participant characteristics

**FIGURE 1:**
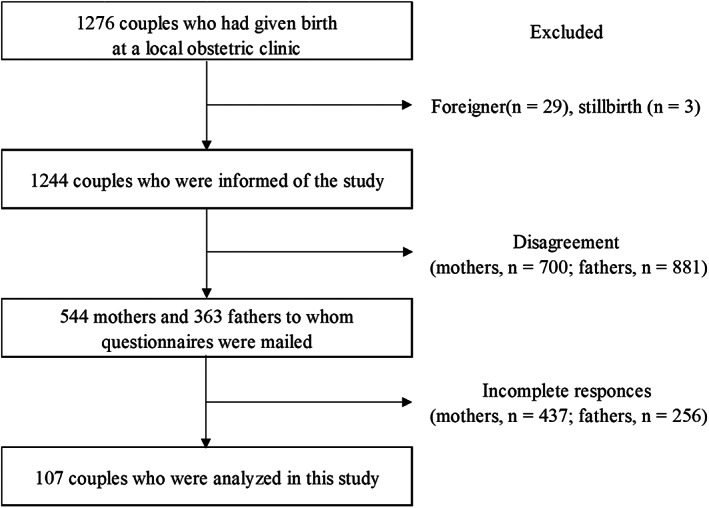
Flowchart of the participants included in the study.

Couples in which one partner was a foreigner (*n* = 29) or the offspring was stillborn (*n* = 3) were excluded from the 1276 couples enrolled. The study informed 1244 couples, and 544 mothers and 363 fathers gave their consent (Figure 1). The number of couples who responded to the survey was 363 (100%) at T1, 176 (48.5%) at T2, 155 (42.7%) at T3, and 137 (37.8%) at T4. At the four time points from T1 to T4, 107 (29.5%) were complete respondent pairs. Overall, data from 107 couples (107 fathers and 107 mothers) were analyzed. Table 1 summarizes participant characteristics. The mean age of fathers was 33.6 ± 5.7 years and that of the mothers was 32.1 ± 4.6 years. Sixty‐six mothers (61.7%) were primiparous. Of the 107 couples, 17 (15.9%) of their infants required follow‐up monitoring. Most fathers (97.2%) were working, and 24.3% of mothers at the first‐year postpartum were working. Only 8 (7.5%) fathers had taken childcare leave. Table 2 shows the prevalence of PPD for fathers and mothers, respectively, at each survey period. The prevalence of paternal PPD was 14.0% at T1, 12.1% at T2, 23.4% at T3, and 17.8% at T4. Whereas the prevalence of maternal PPD was 6.5% at T1, 7.5% at T2, 7.5% at T3, and 8.4% at T4.

**TABLE 1:** Participants characteristics.

	Fathers (*n* = 107)	Mothers (*n* = 107)
Age, mean (SD), years	33.6 (5.7)	32.1 (4.6)
Family structure^a^		
Nuclear, *n* (%)	94 (88.0)	
Joint, *n* (%)	12 (11.2)	
Parity		
Primiparous, *n* (%)	66 (61.7)	
Multiparous, *n* (%)	41 (38.3)	
Child health status^a^		
Good, *n* (%)	89 (83.2)	
Monitor progress, *n* (%)	17 (15.9)	
Employment status^b^		
Employed, *n* (%)	104 (97.2)	26 (24.3)
Unemployed, *n* (%)	3 (2.8)	81 (75.7)
Paternal childcare leave^a^		
Taking, *n* (%)	8 (7.5)	
Not taking, *n* (%)	97 (90.7)	

Abbreviation: SD, standard deviation.

^a^ Missing values on family structure (*n* = 1), child health status (*n* = 1), and paternal childcare leave (*n* = 2).

^b^ Employment status of the mother indicates 1 year postpartum.

**TABLE 2:** Prevalence of postpartum depression for fathers and mothers.

		T1	T2	T3	T4
Fathers	EPDS < 8, *n* (%)	92 (86.0)	94 (87.9)	82 (76.6)	88 (82.2)
	EPDS ≧ 8, *n* (%)	15 (14.0)	13 (12.1)	25 (23.4)	19 (17.8)
Mothers	EPDS < 9, *n* (%)	100 (93.5)	99 (92.5)	99 (92.5)	98 (91.6)
	EPDS ≧ 9, *n* (%)	7 (6.5)	8 (7.5)	8 (7.5)	9 (8.4)

Abbreviations: EPDS, Edinburgh Postnatal Depression Scale; T1, 5th day after birth; T2, 1 month after birth; T3, 6 months after birth; T4: 1 year after birth.

### 3.2 Assessment scale at each survey period and their relationship to the EPDS

Table 3 shows the trends in scores over the survey period. EPDS and SOC scores did not differ according to survey period for either fathers (EPDS, *p* = 0.056; SOC, *p* = 0.518) or mothers (EPDS, *p* = 0.143; SOC, *p* = 0.298). QMI showed significant differences between survey periods for both fathers (*p* = 0.001) and mothers (*p* < 0.001). QMI in fathers was significantly lower at T2 (*p* = 0.040) and T4 (*p* = 0.003) than at T1, and QMI in mothers was significantly lower at T4 than at T1 (*p* = 0.001), T2 (*p* = 0.011), and T3 (*p* = 0.045). SSS in fathers and MIBS in mothers also differed significantly according to the survey period (SSS, *p* < 0.001; MIBS, *p* = 0.004). SSS in fathers was significantly lower at T1 than at T2 (*p* = 0.001), T3 (*p* = 0.001), and T4 (*p* < 0.001). MIBS in mothers was higher at T1 than at T2 (*p* = 0.021) and T3 (*p* = 0.043).

**TABLE 3:** Comparison of each assessment scale by survey period.

	Fathers (*n* = 107)		Mothers (*n* = 107)	
	M (SD)	*p*‐value^a^	M (SD)	*p*‐value^a^
EPDS		0.056		0.143
T1	4.22 (2.65)		3.22 (3.03)	
T2	3.79 (3.07)		3.54 (3.18)	
T3	4.58 (3.45)		3.96 (2.91)	
T4	4.01 (3.67)		3.56 (3.81)	
SOC		0.518		0.298
T1	65.1 (11.5)		68.1 (12.2)	
T2	65.2 (12.7)		67.9 (11.8)	
T3	66.2 (12.9)		67.8 (11.9)	
T4	65.1 (12.8)		66.5 (14.2)	
QMI		0.001		<0.001
T1	22.0 (2.5)	T1 > T2, *p* = 0.040	21.9 (2.5)	T1 > T4, *p* = 0.001
T2	21.1 (3.3)	T1 > T4, *p* = 0.003	21.3 (3.4)	T2 > T4, *p* = 0.011
T3	21.5 (3.0)		21.1 (3.6)	T3 > T4, *p* = 0.045
T4	20.9 (3.1)		20.4 (4.3)	
SSS		<0.001		
T1	17.0 (4.3)	T1 < T2, *p* = 0.001		
T2	18.6 (4.6)	T1 < T3, *p* = 0.001		
T3	18.4 (4.6)	T1 < T4, *p* < 0.001		
T4	18.8 (4.5)			
MIBS				0.004
T1			1.5 (1.8)	T1 > T2, *p* = 0.021
T2			1.0 (1.5)	T1 > T3, *p* = 0.043
T3			1.0 (1.5)	
T4			1.3 (1.8)	

Abbreviations: EPDS, Edinburgh Postnatal Depression Scale; MIBS, Mother‐to‐Infant Bonding Scale; SD, standard deviation; SOC, sense of coherence; SSS, Social Support Scale; QMI, Quality Marriage Index. T1, 5th day after birth; T2, 1 month after birth; T3, 6 months after birth; T4: 1 year after birth.

^a^ Repeated measure ANOVA with the Bonferroni correction for multiple comparisons.

Table 4 shows the correlations between EPDS scores and each of the assessment score for fathers and mothers, respectively. In fathers, SOC and QMI were significantly correlated with EPDS at all survey periods. SSS was significantly correlated with EPDS at T2, T3, and T4. In mothers, SOC and MIBS were significantly correlated with EPDS at all survey periods. QMI was significantly correlated with EPDS at T2, T3, and T4.

**TABLE 4:** Correlations between EPDS score and each assessment scale by survey period.

	Father's EPDS				Mother's EPDS			
	T1	T2	T3	T4	T1	T2	T3	T4
Father's EPDS score					0.006	−0.046	0.084	−0.047
Mother's EPDS score	0.006	−0.046	0.084	−0.047				
SOC score	−0.583***	−0.540***	−0.754***	−0.690***	−0.650***	−0.645***	−0.577***	−0.677***
QMI score	−0.310**	−0.361***	−0.578***	−0.430***	0.145	−0.214*	−0.340***	−0.465***
SSS score	0.111	0.357***	0.244**	0.401***				
MIBS score					0.497***	0.511***	0.489***	0.554***

Abbreviations: EPDS, Edinburgh Postnatal Depression Scale; SOC, sense of coherence; SSS, Social Support Scale; T1, 5th day after birth; T2, 1 month after birth; T3, 6 months after birth; T4: 1 year after birth; QMI, Quality Marriage Index.

*Note*: Pearson's correlation analysis *p < 0.05, **p < 0.01, ***p < 0.001.

### 3.3 The related factors of PPD

Table 5 shows the results of multiple regression analysis for fathers and mothers, respectively. SOC was the strongest factor for EPDS throughout all survey periods in fathers (T1, *β* = −0.580; T2, *β* = −0.442; T3, *β* = −0.629; T4, *β* = −0.563). QMI and SSS were significant factors for EPDS in some survey periods (QMI, T1, *β* = −0.180; T3, *β* = −0.243; SSS, T2, *β* = 0.178). Similarly in mothers, SOC was the strongest factor for EPDS throughout all survey periods (T1, *β* = −0.497; T2, *β* = −0.496; T3, *β* = −0.382; T4, *β* = −0.428). QMI and MIBS were significant factors for EPDS in some survey periods (QMI, T4, *β* = −0.251; MIBS, T1, *β* = 0.173; T2, *β* = 0.271; T3, *β* = 0.294).

**TABLE 5:** Results of multiple regression analysis for father's and mother's EPDS.

	Father's EPDS				Mother's EPDS			
	T1	T2	T3	T4	T1	T2	T3	T4
	*β*	*β*	*β*	*β*	*β*	*β*	*β*	*β*
Father's EPDS					0.052	−0.010	0.139	−0.154
Mother's EPDS	−0.051	−0.147	0.000	−0.128				
SOC score	−0.580**	−0.442**	−0.629**	−0.563**	−0.497**	−0.496**	−0.382**	−0.428**
QMI score	−0.180	−0.108	−0.243**	−0.076	0.133	−0.062	−0.167	−0.251**
SSS score	−0.166	0.178*	−0.029	0.161				
MIBS score					0.173*	0.271**	0.294**	0.182
Sociodemographic and Obstetrical Data								
Age	0.105	0.159	0.146*	0.040	−0.128	−0.061	−0.112	−0.087
Family structure (Nuclear = 0, Joint = 1)	−0.059	0.093	−0.037	0.092	0.025	0.022	−0.009	0.012
Parity (Primiparous = 0, Multiparous = 1)	−0.073	−0.062	0.018	0.019	−0.159	0.056	0.011	0.037
Child health status (Good = 0, Monitor progress = 1)	−0.127	−0.203*	−0.107	−0.006	0.066	−0.120	−0.021	0.006
Employment status (Unemployed = 0, Employed = 1)	−0.138	−0.033	−0.010	−0.081	−0.048	−0.003	−0.007	−0.013
Paternal childcare leave (Not taking = 0, Taking = 1)	−0.027	−0.020	−0.173**	−0.073	0.011	−0.019	0.005	0.077
*R* ^*2*^	0.427	0.449	0.691	0.524	0.531	0.483	0.423	0.498

Abbreviations: EPDS, Edinburgh Postnatal Depression Scale; SOC, sense of coherence; SSS, Social Support Scale; T1, 5th day after birth; T2, 1 month after birth; T3, 6 months after birth; T4, 1 year after birth; QMI, Quality Marriage Index.

*Note*: Multiple regression analysis (forced entry method) using father's and mother's EPDS as dependent variables.*p < 0.05, **p < 0.01.

Table 6 shows the association between the values of the rating scale measured at T1 and the EPDS measured after that. EPDS at T2, T3, and T4 in fathers was significantly associated with SOC at T1. Conversely, mothers' EPDS at T2 and T3 were significantly associated with SOC at T1.

**TABLE 6:** Relationship between assessed values at T1 and subsequent EPDS.

	Father's EPDS			Mother's EPDS		
	T2	T3	T4	T2	T3	T4
	*β*	*β*	*β*	*β*	*β*	*β*
Father's EPDS (T1)				−0.051	−0.037	−0.105
Mother's EPDS (T1)	−0.232*	−0.171	−0.200*			
SOC score (T1)	−0.418**	−0.500**	−0.517**	−0.506**	−0.407**	−0.170
QMI score (T1)	−0.144	−0.207*	−0.141	−0.043	−0.083	−0.268*
SSS score (T1)	−0.025	0.004	−0.005			
MIBS score (T1)				−0.027	−0.131	−0.051
Sociodemographic and Obstetrical Data						
Age	0.245**	0.249**	0.136	−0.052	−0.094	−0.249**
Family structure (Nuclear = 0, Joint = 1)	0.146	0.030	0.083	−0.006	0.025	0.007
Parity (Primiparous = 0, Multiparous = 1)	−0.163	−0.067	−0.060	0.065	0.033	0.033
Child health status (Good = 0, Monitor progress = 1)	−0.143	−0.062	−0.037	−0.129	0.069	0.074
Employment status (Unemployed = 0, Employed = 1)	−0.052	0.038	−0.115	−0.021	0.026	0.013
Paternal childcare leave (Not taking = 0, Taking = 1)	−0.049	−0.060	−0.004	−0.003	0.002	0.033
*R* ^*2*^	0.382	0.486	0.404	0.256	0.168	0.190

Abbreviations: EPDS, Edinburgh Postnatal Depression Scale; SOC, sense of coherence; SSS, Social Support Scale; T1, 5th day after birth; T2, 1 month after birth; T3, 6 months after birth; T4, 1 year after birth; QMI, Quality Marriage Index.

*Note*: Multiple regression analysis (forced entry method) using father's and mother's EPDS as dependent variables. *p < 0.05, **p < 0.01.

## 4 DISCUSSION

This longitudinal study found that 12.1%–23.4% for fathers and 7.5%–8.4% for mothers showed signs of PPD during the first year after delivery. Furthermore, SOC was associated with EPDS scores for both fathers and mothers at all survey periods.

The prevalence of paternal PPD at 6 and 12 months in this study was slightly higher than in other Japanese studies.^17^, ^29^ According to a meta‐analysis of the prevalence of paternal PPD in Japan, most studies using the EPDS were conducted within the 6‐month postpartum period.^5^ That meta‐analysis suggests that the prevalence of paternal PPD peaks at 3–6 months after delivery. In a Japanese longitudinal study using the Center for Epidemiologic Studies Depression Scale, the highest prevalence of paternal PPD was at 1 year postpartum.^43^ Our findings suggest that PPD is more common in fathers and that we need to pay particular attention to the occurrence of paternal PPD during the 6 months to 1‐year postpartum period. The Japanese government is gradually implementing legislation starting in April 2022 to encourage fathers to take postnatal parental leave.^44^ Managing PPD requires the development of social initiatives that include support for fathers as well as mothers.

Among the factors we studied, SOC has the strongest association with PPD for both fathers and mothers throughout the first year after delivery. In addition, SOC in the initial few days postpartum predicted parents' EPDS during most of the subsequent study period. Several previous studies have reported associations between SOC and postpartum emotional disorders, suggesting that SOC may be protective.^18^, ^19^, ^20^, ^22^ Iwanowicz‐Palus et al. showed that higher levels of SOC are associated with lower levels of EPDS after delivery. SOC refers to an individual's ability to perceive and respond to stressful situations and to demonstrate confidence in their ability to manage the situation through social support and other resources.^45^ While life events such as pregnancy and childbirth are joyful and happy for many fathers and mothers, these are also stressors.^46^ During the transition to parenthood, stress levels increase as parents face major changes in roles, relationships, and lifestyles.^47^, ^48^ Successfully coping with those stressors is important to prevent PPD. SOC has also been reported to increase an individual's effectiveness in coping with stressors^21^ and is an important factor in preventing postpartum emotional disturbances. Furthermore, previous study not related to childbirth indicate that interventions may be able to increase SOC.^49^ Our findings suggest that developing strategies that enhance SOC may play a vital role in managing PPD for both fathers and mothers.

Although the period of influence on EPDS was limited, QMI for both fathers and mothers, MIBS for mothers, and SSS for fathers were found to be associated with EPDS. Previous studies have shown that marital satisfaction is associated with PPD for both fathers and mothers.^27^, ^50^ It has also been found that mother‐infant bonding is a predictor of psychological distress in mothers^24^ and that social support is related to paternal PPD.^16^ However, given the bidirectional association between these psychosocial factors and postpartum depression, the relationship may be more complex. For instance, disturbances in mother‐infant bonding in the first year postpartum are also predicted from having PPD.^25^ Our findings suggest that factors related to PPD may differ depending on the postpartum period, underscoring the need to sort out the complexity of these relationships according to period.

Several limitations should be considered in interpreting our results. First, we included couples in which both mothers and fathers completed questionnaires at all four time points, hence the low response rate. Assuming that parents who understood the purpose of this study and had the mental and financial resources to respond with a high awareness of childcare, the EPDS for non‐respondents could be high. However, future research is needed to clarify this. The family environment, including mental and financial concerns, is also crucial for PPD.^29^ In addition, since the present study was conducted in a single clinic located in an urban area, the findings need to be validated in future studies with a larger and more diverse sample. Second, we assessed PPD based on a self‐reported EPDS score rather than a full diagnostic procedure. However, since the EPDS is a validated and reliable instrument that is frequently used worldwide, the risk of misclassification bias is expected to be small. Third, residual confounders may have occurred from unmeasured confounders such as sociodemographic variables including income and educational history and parental mental status during the pregnancy. Finally, the present study was conducted using data obtained before the COVID‐19 pandemic. Social restrictions and loss of social support associated with COVID‐19 may be associated with PPD.^51^ Women with perinatal psychological problems and those who experienced emotional support may be positively affected by less external stimuli due to lockdown restrictions.^52^ Therefore, the social context of COVID‐19 should be considered when interpreting our results. Additionally, the situation surrounding maternal and child health care has been pointed out as declining birthrates, late marriages and childbearing, rising unmarried rates, the shift to nuclear families, isolation of childcare, and child abuse.^12^ Systems have been developed to enable parents to take childcare leave in cooperation.^44^ It is necessary to target support not only to mothers and their children but also to parents and their children, including fathers. In particular, support for fathers is also essential, as we live in an era where men are required to take parental leave. Despite these limitations, this is a significant one‐year longitudinal study investigating psychosocial factors for parents and underscores the need for interventions for psychosocial factors, including SOC just after birth and at each point.

## 5 CONCLUSIONS

This longitudinal study was conducted from a few days to 1 year after delivery to investigate the factors affecting PPD in fathers and mothers during each survey period. Our findings suggest that SOC is the strongest predictor of PPD for both fathers and mothers throughout the first year after delivery. Developing strategies to enhance stress coping skills may be important to prevent PPD in both fathers and mothers.

## AUTHOR CONTRIBUTIONS

Y.Y., M.M, Y.H., and G.T. designed the study; Y.Y. collected data; Y.Y., M.M., Y.H., A.T., R.I., S.H., A.I., and G.T. analyzed data and wrote the manuscript. All authors contributed to the intellectual content of this manuscript and approved the final manuscript as submitted.

## FUNDING INFORMATION

This study was supported by a Grant‐in‐Aid for Scientific Research from the Ministry of Education, Culture, Sports, Science, and Technology, Japan (22592485).

## CONFLICT OF INTEREST STATEMENT

The authors declare no conflict of interest.

## ETHICS STATEMENT

Approval of the research protocol by an institutional reviewer board: The study protocol was approved by the Saga University (Faculty of Medicine) Ethics Committee (Ref. Nos. 23–60).

Informed consent: All informed consent was obtained from the participants.

Registry and the registration no. of the study/trial: N/A.

Animal studies: N/A.

## Data Availability

The data that support the findings of this study are not publicly available due to ethical restrictions. Specifically, informed consent for public data release was not obtained from the participants.
